# Identifying hotspots in land use land cover change and the drivers in a semi-arid region of India

**DOI:** 10.1007/s10661-018-6919-5

**Published:** 2018-08-20

**Authors:** Vijayasekaran Duraisamy, Ramkumar Bendapudi, Ajit Jadhav

**Affiliations:** Centre for Resilience Studies, Watershed Organisation Trust, Pune, 411009 India

**Keywords:** India, Land use land cover, Remote sensing, GIS, Semi-arid, Drivers of change

## Abstract

The study examines long-term land use/land cover change (LUCC) at a finer scale in a semi-arid region of India. The objectives were to study and quantify the spatiotemporal LUCC and uncover the major drivers causing the change in the Mula Pravara river basin, which is located in a semi-arid region of Maharashtra state, India. Advanced very high-resolution radiometer (AVHRR)-Normalized Difference Vegetation Index (NDVI) 3g data for the years 1982–2015 were used to identify the ‘hotspot’ with significant positive and negative LUCC. Multi-temporal Landsat imagery was used to produce finer scale land use maps. From 1991 to 2016, the agricultural land area increased by approximately 98% due to the conversion of uncultivable and fallow lands to agriculture. The built-up area increased by 195%, and in recent years, an urban expansion has occurred in agricultural lands close to the urban fringe areas. There has been a shift from food crops to commercial crops, as observed from the steep increase in the amount of land under horticultural plantations, by 1601% from 2001 to 2016. In addition, the area under forest canopy was reduced in the protected regions. Institutional factors that improved access to water resources were the major drivers of change in hotspots, especially in the context of agriculture. Technological and economic factors were the other supporting factors that contributed to the change. This study demonstrates the advantages of using satellite remote sensing techniques to monitor the LUCC, which is useful for predicting future land changes and aids in planning adaptation strategies.

## Introduction

Land use/land cover change (LUCC) is the major underlying cause of global environmental change (Sala 2000). Present land use practices are more focused on satisfying short-term supply needs for a growing population without considering the long-term loss in ecosystem services and environmental damage. A change in land use is one of the nine “planetary boundaries”, and humanity may soon approach the boundary that jeopardizes the safe operating space for humanity with respect to the earth system process (Rockström et al. 2009). The “human-domination” model (land transformation, biotic addition, and losses) alters the earth’s bio-geochemistry and influences climate change, resulting in a loss of biological diversity (Ojima et al. 1994; Vitousek et al. 1997). The arid and semi-arid parts are most vulnerable to climate change, where resources are limited and the adaptive capacity is low (Ostwald and Chen 2006; Gupta et al. 2011; El-Beltagy et al. 2012). The rapid LUCC in these regions can exacerbate the effect of climate change and contribute to resource overuse, land degradation, drought and flood hazards, and loss of livelihoods (Watson et al. 1997; IPCC 2001; El-Beltagy et al. 2012). The detrimental effects of unplanned and ad hoc LUCC can be reduced through suitable land management strategies, which require information on the present and future scenarios of LUCC. Studies on the global LUCC started in the late 1990s with the International Geosphere-Biosphere Programme (IGBP) and the International Human Dimensions Programme (IHDP) of Global Environmental Change (Turner et al. 1994; Uhrqvist and Lövbrand 2014). The myriad data gaps in global land cover information in IGBP were well-addressed using satellite data (Loveland et al. 1991). Over the last three decades, the availability of global satellite data products have aided in the development of spatially explicit global and regional land cover databases at various spatial resolutions, including 1 km to 30 m (Grekousis et al. 2015). However, many of these existing databases do not support change detection because they are of different spatial scales and lack multi-temporal consistency, which increases the uncertainty in the land change analysis. The AVHRR NDVI data are adequately used in national and regional scale studies to identify vegetation dynamics (Singh et al. 2003; Hansen and DeFries 2004; Bégué et al. 2011; Duan et al. 2011) and land cover changes (Steyaert et al. 1997; Thenkabail et al. 2007). Landsat series satellite data are used in finer scale land use mapping studies (Peiman 2011; Nutini et al. 2013; Walters 2017). Combining the coarse-scale data such as AVHRR with finer scale Landsat data helps to address the spatial heterogeneity and explains the significant processes and factors involved in land system change.

LUCC is driven by complex proximate (direct) causes based on local level decisions and underlying (in-direct) driving forces at the regional and national levels (Lambin et al. 2001). The underlying driving forces influencing the proximate causes were categorized into biophysical, economic, technological, demographic, institutional, and cultural factors (Geist and Lambin 2002). These factors vary with scale and are often simplified as “cause-consequence relationships” at the global scale (Lambin et al. 2001). Global scale studies may influence decisions at the international scale but can only be effective at influencing finer scale decisions if they are coupled with finer scale analysis (DeFries et al. 2012). In recent years, regional and local level studies have started exploring LUCC along with its various “causes” and “underlying forces” (Ostwald et al. 2009; Qasim et al. 2013; Plieninger et al. 2016) to recognize the complexity of land use changes.

The overall objective of the present study is to estimate and examine the finer scale LUCC over the last three decades using Landsat satellite imagery in the Mula-Pravara River basin located in a semi-arid region of India; to identify the hotspots of LUCC and investigate driving forces that cause change.

## Study area

**Fig. 1:**
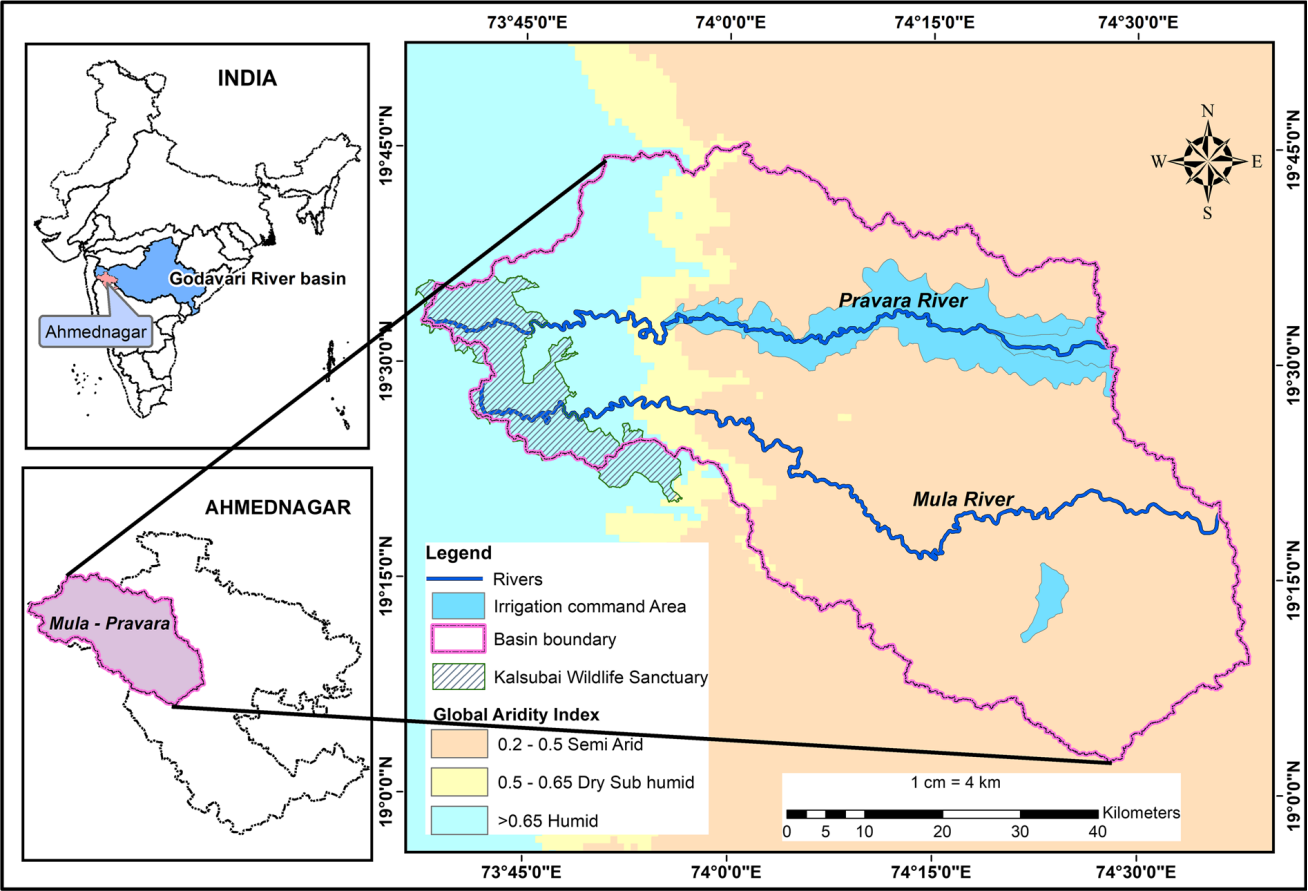
Location map of the study area, Mula-Pravara basin

The study area is part of the upper Godavari River basin and lies between 73° 37′ 20″–74° 38′ 33″ E and 19° 2′ 20″–19° 45′ 31″ N, covering a geographical area of 4303 km^2^ in Ahmednagar district, Maharashtra, India. The Mula and Pravara Rivers originate in Western Ghats (WG) (the hills of Akole) and flowing eastwards, and they are notable perennial right bank tributaries of the Godavari (Central Water Commission 2015). The administrative divisions of Akole, Sangamner, and Parner cover the majority of the study area (Fig. 1).

According to the global aridity index (Zomer et al. 2008), almost 75% of the Mula-Pravara is semi-arid, and the remaining portion has humid climatic conditions (Fig. 1). The basin is situated in a rain shadow region. The mean annual rainfall varies from approximately 508 mm in the highlands of Akole to 416 mm in Sangamner, and the total number of rainy days is less than 40. The WG region of the study area receives comparatively more rainfall (highly variable) (Fig. 8), and it diminishes considerably in the eastern part. The mean minimum and maximum temperatures are 18 °C and 32 °C, respectively. The forest cover type in this region is “2A/C2-Southern Tropical Semi-evergreen” in the west and “5A/C3-Southern Tropical Dry Mixed Deciduous” in the eastern side, which corresponds to the amount of rainfall (Prakash 2010). Agriculture is the major livelihood in Mula-Pravara and accounts for 46% of the total geographical area. Crops are cultivated during three seasons: (i) Kharif (July–October, monsoon crops), (ii) Rabi (October–March, winter crops), and (iii) Zaid (March–June, summer crops). Drought occurs very frequently and is noted once every 3 years in this region (Gupta et al. 2011). Agriculture is affected due to the occurrence of dry spells (for a duration of 2–10 weeks per year), the delayed onset of the monsoon, and the early cessation of the monsoon (Government of Maharashtra 2004). In the most recent national groundwater assessment, the central part of the basin in the *Sangamner* administrative block has been categorized as an “overexploited” groundwater zone (Central Groundwater Board 2017). The land use/land cover (LULC) of Mula-Pravara experienced dramatic changes and a noticeable expansion in the agricultural area, along with shifts towards commercial crops such as soybeans, sugarcane, and pomegranates (Chandra and Tarachand 2010; Rede et al. 2013), which put farmers at greater risk in the future. Thus, the Mula-Pravara basin was selected to study the major land transitions, hotspots of LUCC, and drivers of change to understand its vulnerabilities and contribute to suitable adaptation plans for future climate risks.

## Materials and methods

The remotely sensed satellite imagery from the United States Landsat series and the AVHRR NDVI 3g time series data were used in this study to identify the finer-scale LUCC and hotspots of change, respectively. The results acquired from the remote sensing analysis were studied in the context of the government policy changes, schemes introduced, and outcomes measured by other studies. Apart from these data, secondary data sources such as government census data pertaining to the area were used to understand the drivers of change.

### Data acquisition

The satellite imagery used in this study is from the United States Geological Survey (USGS) earth explorer (https://earthexplorer.usgs.gov). The study area is covered by Landsat frame path row 147-46 and 147-47 of the Worldwide Reference System (WRS). The multi-date multi-sensor satellite imagery was collected for successive cropping seasons during the study years. In total, 24 scenes from Landsat 4–5 Thematic Mapper (TM), Landsat 7 Enhanced Thematic Mapper Plus (ETM+), and Landsat 8 Operational Land Imager (OLI) were used to cover the study years 1991, 2001, 2011, and 2016 (Table 1).

**Table 1:** List of satellite imagery used in this study

Study year	Season	Acquisition date	Sensor	Path/row	Resolution (m)
1991	Kharif	21-Sep-91	Landsat 4–5 TM	147/46, 47	30
	Rabi	12-Dec-89	Landsat 4–5 TM	147/46, 47	30
	Summer	03-Apr-90	Landsat 4–5 TM	147/46, 47	30
2001	Kharif	5-Oct-01	Landsat 7 ETM^+^	147/46, 47	30
	Rabi	25-Jan-00	Landsat 4–5 TM	147/46, 47	30
	Summer	4-Apr-99	Landsat 7 ETM^+^	147/46, 47	30
2011	Kharif	27-Sep-09	Landsat 4–5 TM	147/46, 47	30
	Rabi	23-Jan-11	Landsat 4–5 TM	147/46, 47	30
	Summer	26-Apr-10	Landsat 4–5 TM	147/46, 47	30
2016	Kharif	1-Oct-15	Landsat 8 OLI	147/46, 47	30
	Rabi	5-Jan-16	Landsat 8 OLI	147/46, 47	30
	Summer	10-Apr-16	Landsat 8 OLI	147/46, 47	30
1982–2015	Bimonthly	1982–2015	AVHRR-GIMMS	–	8000

The Landsat satellite sensor details and the wavelengths of the spectral bands are available at https://landsat.usgs.gov/what-are-band-designations-landsat-satellites. The acquisition of cloud-free satellite imagery is a major limitation in tropical regions. Therefore, the dates close to the study year with cloud-free imagery were used. The acquired scenes are level-1 terrain-corrected (i.e., the relief displacements are corrected using the ground control points and the digital elevation model), which are most suitable for pixel-level time series studies. The corrected images were provided in GeoTiff format and resampled to 30 m with the Universal Transverse Mercator (UTM)-World Geodetic System (WGS) 84 projection using the cubic convolution method.

**Fig. 2:**
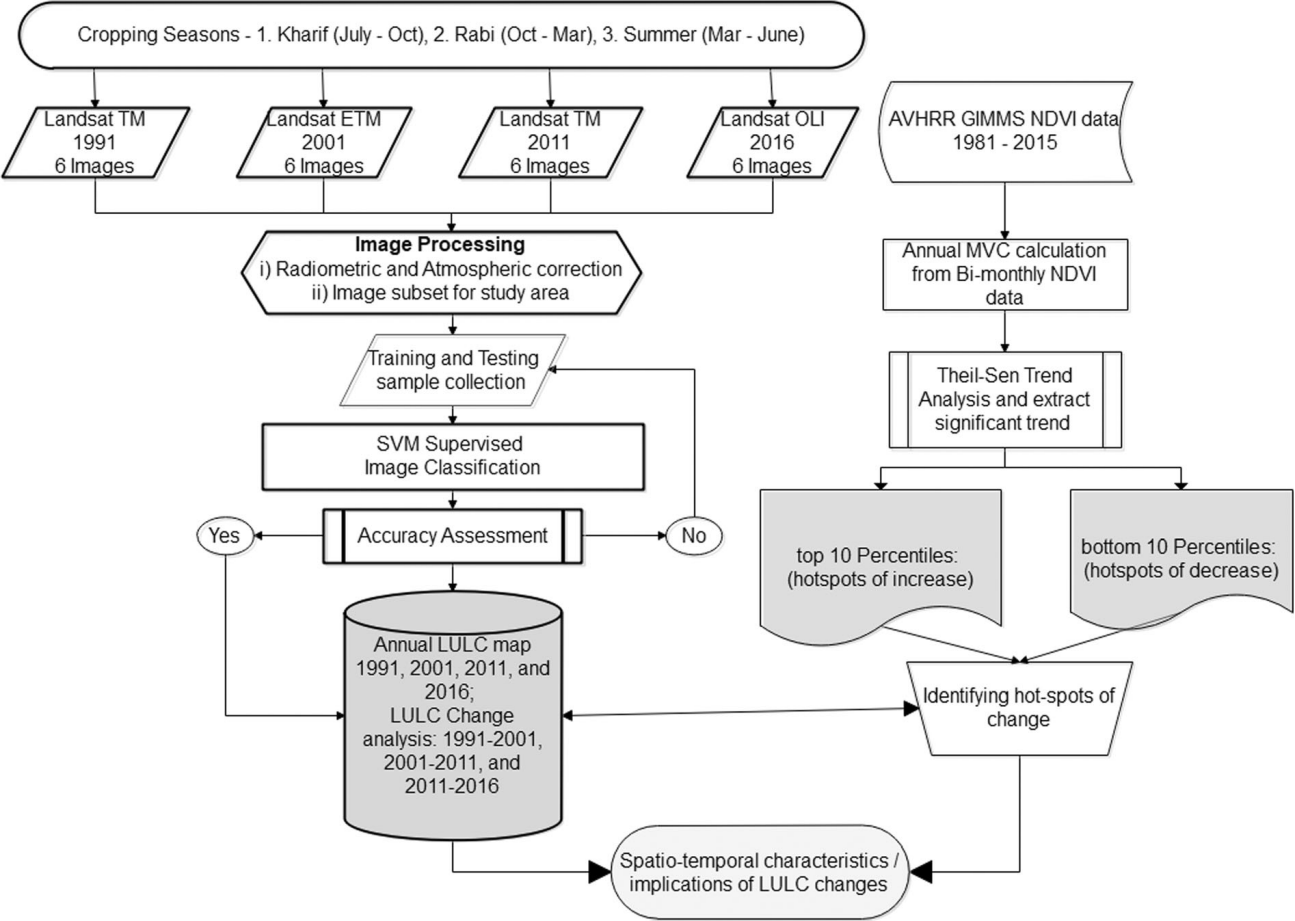
Stepwise methodology used in this study

In addition, the AVHRR-NDVI 3g data processed by NASA’s ECOCAST lab (Pinzon and Tucker 2014) were used in this study. The bi-monthly composite NDVI data with 8 km × 8 km spatial resolution were collected for the 1982–2015 time period. These are the global mosaic data available with the Albers Conical Equal Area projection. These data were used to identify the LUCC hotspots with respect to the changes in vegetation. The LUCC “hotspots” were identified by analyzing the significant trends in vegetation greening and browning from the time-series NDVI 3g data as proposed by Guay et al. (2014). To mask the forest area from other vegetation classes, the designated forest boundary delineated from the Survey of India topographical sheets at the 1:50,000 scale was used. The forest area was classified separately to avoid confusion with other vegetation classes. In our study area, agriculture is a major land use covering 46% (the year 2016) of the landscape. Because we are using seasonal satellite imagery, the agricultural classes are segregated as single crop-rain-fed (SC-R), single crop-Rabi and summer (SC-R+S), double crop (DC), triple crop (TC), fallow land (FL), and plantations (PL). This segregation will be useful for studying the seasonal cropping changes efficiently. The handheld GPS Garmin-eTrex® 20× with 3 m of location accuracy was used to collect ground truth (GT) samples. A total of 294 GT points of agricultural land classes were collected during December of 2016. GT data collected from the respective farmers on location included the types of crops grown during the different seasons (Kharif, Rabi and summer) of 2015–2016 and the source of irrigation. Another 350 samples were collected for other LULC classes using Google Earth™ to assess the classification accuracy. A supervised image classification was performed on Landsat seasonal data to map the finer scale changes. The seasonal image classification results of individual years were combined to produce annual land use maps, and a “post-classification comparison” method for change detection was performed to identify the transitions in various LUCC categories (Fig. 2).

### Image processing

The normalization of seasonal effects is an important component for the time series mapping of land changes from multi-sensor multi-temporal satellite imagery. This approach can help researchers to discriminate between the product artifacts and the changes in the earth process being monitored (Roy et al. 2002). Image processing was performed through a series of sequential operations such as calibration to radiance, atmospheric correction or normalization, image registration, geometric correction, mosaicking, sub-setting, and masking as suggested by Coppin et al. (2004). The image processing and classification were performed using ENvironment for Visualizing Images® (ENVI) remote sensing software. The initial calibration was performed to convert the digital numbers (DN) of the Landsat scenes into absolute radiance units (W m^−2^ sr^−1^) using the scene metadata. The “Fast Line-of-sight Atmospheric Analysis of Hypercubes” (FLAASH) module was used to perform the atmospheric correction (Cooley et al. 2002). The radiance image, tropical atmospheric profile, and rural aerosol model were inputs to the FLAASH. The atmospherically corrected Landsat scenes were seamlessly mosaicked based on the corresponding date and path/row. Sub-setting was performed to extract the area of interest.

### Classification scheme and training sample collection

Based on the existing land use distribution of the Ahmednagar district, the study area was initially categorized into seven broad LULC classes, specifically, agricultural land, built-up land, fallow land, forest, plantations, un-cultivable and cultivable wasteland (UCW), and water bodies. In this study, the seasonal changes in agriculture were also taken into consideration. The seasonal LULC outputs were combined to create an annual composite for the study years. In the annual composites, the agricultural land was divided into four subclasses, SC-R, SC-R+S, DC, and TC. The forest class was divided into three subclasses, dense forest (DF), open forest (OF), and scrub forest (SF), for a better understanding of the transitions and degradations in the forest canopy. The training samples for image classification were obtained from GE™ historical high-resolution imagery. GE provided reference images from 2001 to 2016, and the reference image for the year 1991 was absent. The existing reference data from 2001 were used to compare the spectral properties and visual interpretation signs of the 1991 imagery to define the LULC classes. The Landsat series satellites have almost similar wavelength bands and spectral characteristics that make sample collection easy.

### Image classification and accuracy assessment

The supervised classification method was chosen to perform the image classification. This method has three stages, training, class allocation, and testing (Mathur and Foody 2008). In the training stage, the region of interest (ROI) for different LULC categories was created in ENVI using GE historical imagery. The collected training samples from corresponding Landsat scenes were used to train the classifier. In the present study, a Support Vector Machine (SVM) classifier was employed to categorize the LULC classes. The “One against One” (Milgram et al. 2006) strategy with an SVM polynomial kernel was used. In land cover classification studies, the SVM outperformed many standard classifiers such as the maximum likelihood classifier, neural network, spectral angle mapper, and minimum distance classifier (Vidhya et al. 2014; Razandi et al. 2015; Vidhya et al. 2017). SVM has the advantage of having an Optimal Separating Hyperplane (OSH) to maximize the margin and to locate the optimal boundaries in high-dimensional feature space (Hsu et al. 2016). In addition, it has the ability to generalize the input data with a limited number of training data and achieve higher classification accuracy (Mountrakis et al. 2011). Accuracy assessments provide a guide to the quality of the map and its fitness for a particular purpose (Foody 2002). The classification results were tested using a confusion error matrix as described by Congalton (1991). The reference data and classified data were cross-tabulated for this purpose. The overall performance of the classification was assessed using the overall accuracy and the “*KAPPA*” statistic of the error matrix, and the individual categories were assessed with the user’s and producer’s accuracy (Story and Congalton 1986). The year 2016’s annual composite classification accuracy was assessed with testing samples from GPS GT points and GE reference data. For the other study years, the seasonal image classification accuracy was assessed using a portion of the training samples selected based on proportionate stratified random sampling.

### Post-classification processing and change detection

The “3 × 3 majority filter” was applied to the Landsat image classification results to eliminate the isolated pixels. The annual land use map was produced from the seasonal classification results of the corresponding years. Categories such as the forest type (DF, OF, and SF) and water bodies are influenced by the seasonality. To overcome this limit, the LULC of the respective categories obtained from monsoon season imagery was used to prepare the final annual map. The change detection was performed by “post-classification comparison” method, and it is the most commonly used method in remote sensing change detection studies. The image classification results of the different time periods are produced independently, and comparisons are performed “pixel by pixel” to detect changes in the LULC (Coppin et al. 2004). The spatial trend in the LULC was analyzed by aggregating the study years into three time periods, namely 1991–2001, 2001–2011, and 2011–2016. The “transition matrix” class was prepared for the time periods, and the “from-to” transitions of various land use categories were identified.

### Hotspot identification

A “Theil-Sen trend” analysis (Theil 1992) was performed on the AVHRR-NDVI 3g data (1982–2015) to identify both positive and negative significant trends in the vegetative cover. The “Mann-Kendall” test was used to assess each trend for statistical significance, regarding the pixels as significant when *P* < 0.05. The trend analysis and assessment were executed using the “*ZYP*” software package (Bronaugh and Werner 2013) in “R” software. Furthermore, the threshold values were applied to the trend analysis output to identify the “hotspots” of the change. The top tenth percentile of trend results were considered as locations of positive change (hotspot of increase) and the bottom tenth percentile were considered as locations of negative change (hotspot of decrease). The finer scale LUCC of the hotspot locations was examined using the Landsat image results.

## Results

### Classification accuracy assessment

In any map-making process, the accuracy assessment is an important component. The quality of the prepared maps is assessed before further analysis. The classification accuracy of LULC maps for the years 1991, 2001, 2011, and 2016 was assessed through a confusion error matrix and Kappa statistic. The overall accuracy for the years 1991, 2001, 2011, and 2016 were 85.5%, 85.1%, 79.1%, and 85.7%, respectively. The Kappa statistics of agreement were 0.82, 0.81, 0.74, and 0.84 for the years 1991, 2001, 2011, and 2016, respectively. In this study, the multi-seasonal satellite imagery was used to identify the LULC class transitions. It was observed that the producer accuracy for the plantation was consistently low during the Kharif season for all the years, with an overall accuracy of 12%. The plantation class tends to mix with the agricultural croplands due to the high vegetative cover during monsoonal cropping periods. To overcome this issue, the plantation class with the average producer accuracy of 60% from summer season imagery is used to prepare the annual LULC map in the year 2011. The Landsat 8 OLI imagery of the year 2016 showed a producer accuracy of 97% and good separation of the plantation class from agricultural land. The vegetation separation improved primarily because of the additional shortwave infrared (SWIR) band and the 12-bit radiometric resolution of the Landsat 8 OLI sensor. A total of 294 ground-truth GPS locations were collected for agricultural classes alone. This information was used for an accuracy assessment of the classified LULC image for 2016. The classification results of the year 2016 imagery showed good producer and user accuracies for a maximum number of classes, except the SC-R+S, DF, and OF classes. The SC-R+S showed a user accuracy of 63% and was predominantly mixed with the DC area. Confusion between the DF and OF classes was observed. The accuracy of different LULC classes increased with the integration of seasonal LULC maps from the study years into the annual LULC map. The final LULC classes of the annual map were labeled by comparing the classes from three seasons.

### LULC mapping

**Fig. 3:**
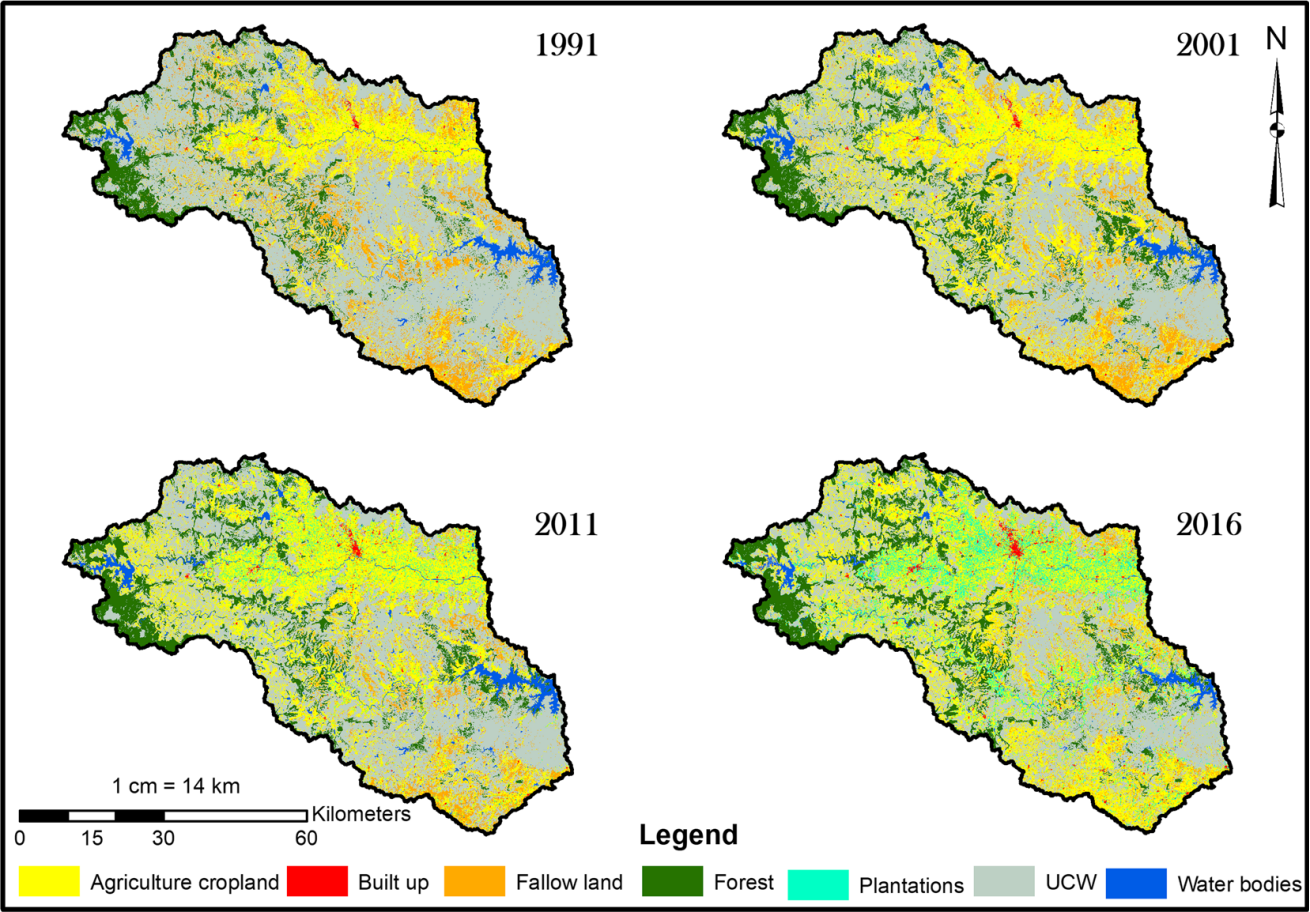
LULC map of Mula-Pravara 1991–2016

The final annual LULC maps for the corresponding years are shown in a collage in Fig. 3. The area statistics and rate of change for the LULC classes over the study period are given in Table 2. The UCW class accounts for the major type of land use in 1991. However, the share decreased from 58% of the total geographical area in 1991 to 38% in 2016. The UCW category includes area under barren land, open scrub, and cultivable wasteland. Agricultural land is the next important land use category, accounting for 30% of the total area in 1991 and increasing to 46% in 2016. From 1991 to 2016, there was a decline in the UCW category by 34.6% and in fallow lands by 60.5%. At the same time, the agricultural land increased by 97.8% (refer to Table 2). The area under horticultural plantations increased dramatically (1601%). Conversions from agricultural cropland to horticultural plantations are illustrated in Fig. 3. There has also been a significant increase in the built-up area during the same period (almost 196%).

**Table 2:** Area and percentage LUCC changes

Categories	Area in (km^2^)				Change (%)			
	1991	2001	2011	2016	1991–2001	2001–11	2011–16	1991–2016
Agriculture	733.13	1150.1	1310.4	1450.7	56.8	13.9	10.7	97.8
Built-up	13.4	19.1	28.4	39.6	43.2	48.1	39.4	195.7
Fallow land	536.3	419.8	278.1	211.8	− 21.7	− 33.7	− 23.8	− 60.5
Forest	393.3	517.4	493.2	561.6	31.5	− 4.6	13.8	42.8
Plantations	19.08	11.19	121.1	324.6	− 41.3	982.1	168.1	1601.4
UCW	2498	2088.6	1932.8	1631.4	− 16.3	− 7.4	− 15.5	− 34.6
Waterbodies	110.2	97.2	139.5	83.7	− 11.7	43.4	− 39.9	− 24
Total	4303.5	4303.5	4303.5	4303.5				

### Land use change detection

The change detection is performed to understand the land transitions and trends in the LUCC. For this purpose, the study years are categorized into three time periods namely, 1991–2001, 2001–2011, and 2011–2016 (Table 2). The post-classification comparison technique was used to calculate the class transitions and rate of change. There has been a steady decline in the area under UCW and fallow lands during each of the three time periods.

**Fig. 4:**
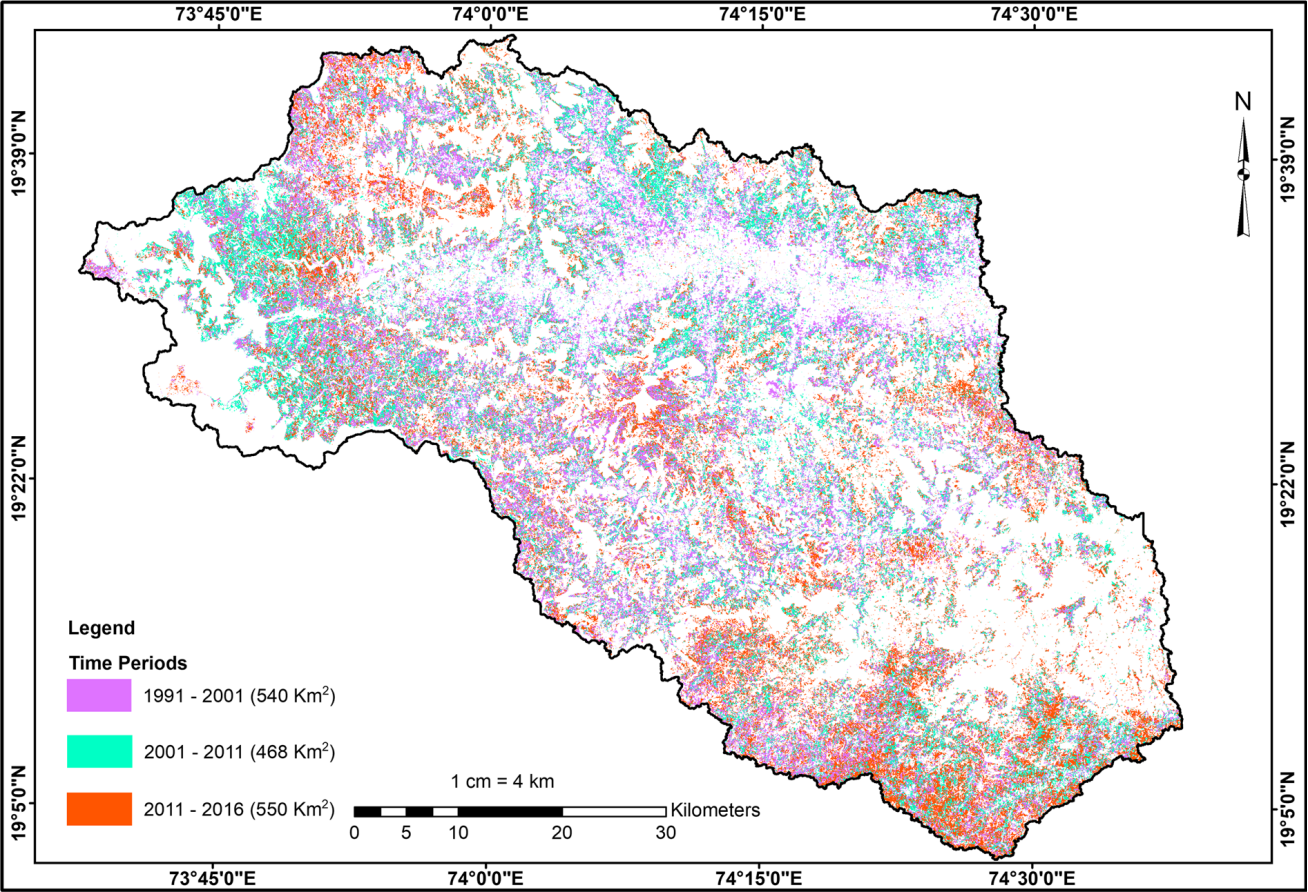
Agricultural land expansion 1991–2016

The area under agricultural land showed a considerable increase from 1991 to 2001 (by almost 57%). The seasonal changes in agriculture (Kharif, Rabi, and Zaid) were also analyzed (Table 3). These analyses show that the area under Kharif in 1991–2001 increased by 61.4%. However, the area under Rabi crops showed a significant increase during the 2011–2016 period (by 60.3%). Some of the reasons could be an increased water-spreading area from watershed development programs, new irrigation projects (e.g., the Nilwande dam irrigation project became operational in 2011 in this region), and an increased dependence on groundwater for irrigation. The area under plantations increased from a mere 11 km^2^ in 2001 to 121 km^2^ in 2011. The area increased to 325 km^2^ in 2016, which is three times that of the area in 2011. The built-up area showed a gradual increase of 43%, 48%, and 39% from 1991 to 2001, 2001–2011, and 2011–2016, respectively. As mentioned earlier, there has been a significant increase in the agricultural land for all the time periods considered. This gain in agricultural land occurred primarily in UCW and fallow lands. The conversion of the UCW category to agricultural land accounted for 355 km^2^ for 1991–2001, 311 km^2^ for 2001–2011, and 400 km^2^ for 2011–2016. Similarly, 185 km^2^, 157 km^2^, and 149 km^2^ of fallow lands were converted into agricultural land for 1991–2001, 2001–2011, and 2011–2016, respectively (refer to Table 4). There is also an increasing trend noted in the agricultural land converted to the cultivable wasteland. Agricultural land amounting to 62 km^2^, 169 km^2^, and 185 km^2^ was converted into cultivable wastelands in 1991–2001, 2001–2011, and 2011–2016, respectively. This type of continuous flux between agricultural land, fallow land, and cultivable wasteland especially occurred in the area where the agriculture was primarily rain-fed (Fig. 4).

**Table 3:** Seasonal cropping area changes in the study area

Agriculture seasons	Area in (km^2^)				1991–01 (%) change	2001–11 (%) change	2011–16 (%) change
	1991	2001	2011	2016			
Kharif	388.8	627.6	678.3	870.4	61.4	8.0	28.3
Rabi	264.2	358.7	399.2	640.1	35.7	11.2	60.3
Zaid	84.2	186.6	341.9	57.7	121.6	83.2	− 83.1

The unchanged agricultural land area increased from 586 km^2^ in 1991–2001 to 826 km^2^ in 2001–2011 (Table 4). Thereafter, there was not much change noted for 2011–2016. All these results imply that the transitions between agriculture lands-UCW-fallow lands could be driven by rainfall patterns, access to irrigation, technological development, and land/water conservation programs. There was a noticeable shift from food crop cultivation to horticulture plantations from 2001 to 2011. Approximately 86 km^2^ of agricultural land was converted into plantations (primarily pomegranate). This conversion continued through 2011–2016, wherein 236 km^2^ agriculture land was brought under plantations (Table 4). This approach might have resulted from the initiatives by the state government to promote pomegranate cultivation under the National Horticulture Mission (NHM) from the year 2005 onwards (Andhra Pradesh productivity council 2013). Pomegranate harvesting occurs throughout the year in Maharashtra and accounts for 70% of the total pomegranate production of India (Chandra and Tarachand 2010).

**Table 4:** LULC transition matrix for the periods 1991–2001, 2001–11, and 2011–16

	1991–2001	Year 2001							
		Agriculture	UCW	Water body	Forest	Built-up	Fallow land	Plantations	1991 total
Year 1991	Agriculture	585.9	62.2	1.3	1.8	1.2	71.5	9.3	733.1
	UCW	355.5	1813.7	11.2	159	3.5	154.7	0.4	2498
	Water body	5.4	18.5	83.6	0	0.3	2.4	0	110.2
	Forest	1.1	53.6	0	338.6	0	0	0	393.3
	Built-up	0	0	0	0	13.4	0	0	13.4
	Fallow land	185.2	140.3	0.8	17.9	0.8	190.6	0.8	536.3
	Plantations	17.1	0.4	0.3	0	0	0.6	0.8	19.1
	2001 total	1150.1	2088.6	97.2	517.3	19.2	419.8	11.2	4303.5
	2001–2011	Year 2011							2001 Total
Year 2001	Agriculture	826.1	169	9.1	1.6	0.6	57.4	86.3	1150.1
	UCW	311.4	1563	30.4	85.9	8	71.6	18.4	2088.6
	Water body	1.2	1.2	93.7	0	0	0.7	0.5	97.2
	Forest	6	102.9	1	405.5	0	1.7	0.3	517.4
	Built-up	0	0	0	0	19.2	0	0	19.2
	Fallow land	156.6	96.6	5.2	0.3	0.6	146.8	13.7	419.8
	Plantations	9.2	0.2	0	0	0	0	1.8	11.2
	2011 total	1310.4	1932.8	139.5	493.2	28.4	278.1	121	4303.5
	2011–2016	Year 2016							2011 Total
Year 2011	Agriculture	813.4	184.7	3	6.5	8.8	57.7	236.3	1310.4
	UCW	400.6	1289.9	4.4	122.7	1.8	76.5	37	1932.8
	Water body	13	37.7	74.4	2.9	0.1	5.9	5.6	139.5
	Forest	3	61.8	0.2	426.7	0	0.1	1.4	493.2
	Built-up	0	0	0	0	28.4	0	0	28.4
	Fallow land	149.5	48.1	1.3	2.9	0.5	66.9	8.9	278.1
	Plantations	71.3	9.2	0.4	0	0	4.7	35.6	121.1
	2016 total	1450.7	1631.4	83.7	561.6	39.6	211.8	324.6	4303.5

Among all the periods considered, the greatest increase in water spreading was observed from 2001 to 2011 (42 km^2^). The water-spread area was reduced by 55.8 km^2^ from 2011 to 2016. The water bodies primarily consisted of reservoirs, lakes and ponds, and water harvesting structures. The built-up area has steadily increased during all the periods. It is important to note that from 2001 to 2011, the area under UCW was converted into built-up land (8 km^2^), whereas from 2011 to 2016, it was primarily the agricultural land that was converted into built-up land (8.8 km^2^).

### Hotspots of change

**Fig. 5:**
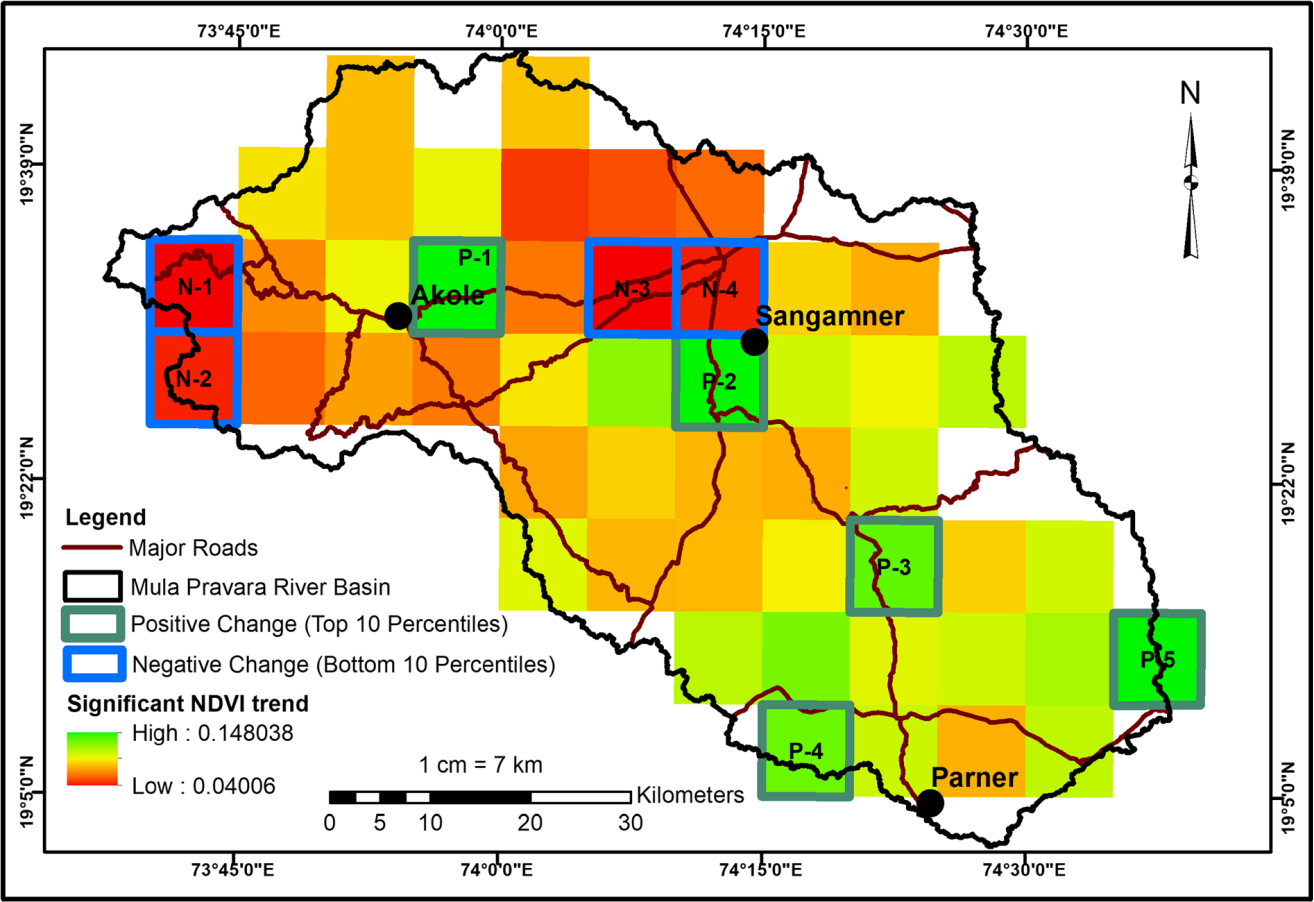
Hotspots of LUCC in Mula-Pravara derived from AVHRR NDVI 3g data

A Theil-Sen trend analysis was performed on NDVI3g data (1982–2015), and it indicated significant trends in vegetative cover (positive and negative changes) throughout the Mula-Pravara basin. The major hotspots of positive (top tenth percentile of increase) and negative changes (bottom tenth percentile of decrease) in vegetative cover were identified (Fig. 5).

**Fig. 6:**
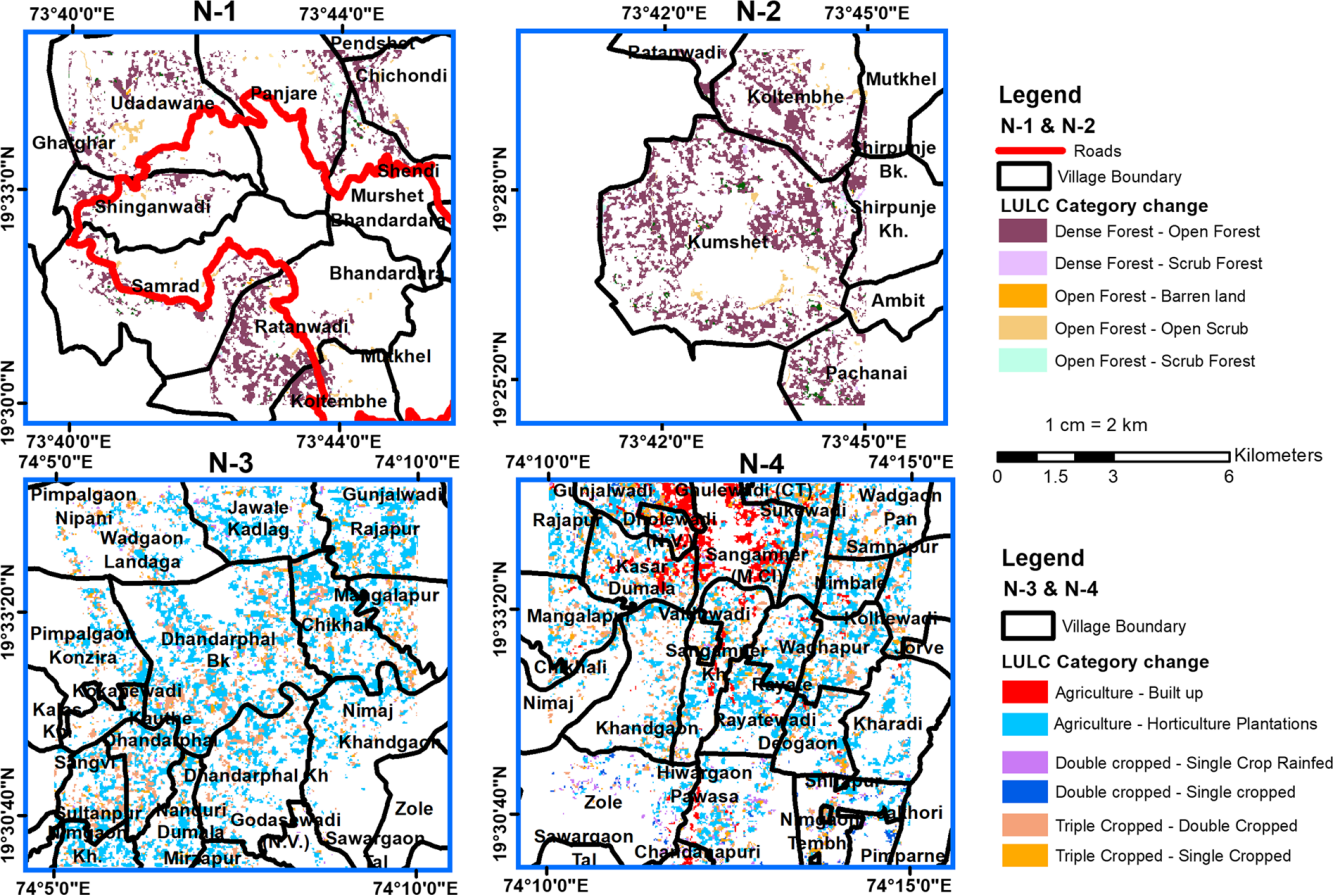
Hotspots of negative change for N1 and N2—forest canopy change, N3, and N4—change in agriculture land

A total of nine hotspots were identified, out of which four indicated negative change and five indicated positive change. The major changes occurred in agricultural land at six hotspots (N3, N4, P1, P2, P3, and P4), and in two hotspots, change occurred in the forest canopy (N1 and N2). The agricultural land showed both positive and negative changes, whereas the forest canopy indicated only negative change. The LULC maps derived from Landsat images (from the years 1991 and 2016) were used to understand the characteristics of land changes in the above-identified hotspots (Figs. 6 and 10). The summary of the changes in hotspots is given in Table 5.

**Table 5:** Characteristics of changes in identified hotspots

Hotspots	Major changes	Location name
1. Negative change in vegetation		
N-1	Change in forest canopy — dense to open	Akole
N-2	Change in forest canopy — dense to open	Akole
N-3	Change in agriculture land 1. Agriculture to horticulture plantation (pomegranate) 2. Triple cropped to double-cropped	Sangamner
N-4	Change in agriculture land — 1. Agriculture to built-up 2. Agriculture to horticulture plantation (pomegranate) 3. Triple cropped to double-cropped	Sangamner
2. Positive change in vegetation		
P-1	Change in Agriculture land — 1. Open scrub to agriculture 2. Fallow land to agriculture 3. Double-cropped to triple cropped 4. Agriculture — plantation (sugarcane)	Akole
P-2	Change in agriculture land — 1. Barren land — agriculture 2. Open scrub to agriculture 3. Double-cropped to triple cropped	Sangamner
P-3	Change in agriculture land — 1. Barren land — agriculture 2. Fallow land to agriculture	Parner
P-4	Change in agriculture land — 1. Open scrub to agriculture 2. Fallow land to agriculture 3. Open scrub — plantation	Parner
P-5	Barren land to open scrub	Parner

## Discussion

### Negative change in vegetation

#### Change in forest canopy

Negative changes were observed in the forest canopies and agricultural lands. The forest canopies were categorized into dense, open, and scrubland based on their respective spectral signatures. The forest cover in this region is “Southern Tropical Semi-Evergreen”. Both N1 and N2 locations experienced significant change from dense forest canopy to open forest canopy, which is consistent with the findings of Panigrahy et al. (2010).

These changes primarily occurred in the designated forest areas of the Kalsubai wildlife sanctuary in the Akole block of the Ahmednagar district. In many locations, encroachment by agricultural activities was observed in N1 and N2 hotspots. Between 2001 and 2016, almost 10.8 km^2^ of forest area was converted into agricultural land (refer to Table 4). The forest degradation was also noticed along the road network of the Bhandardara reservoir in N1 (Fig. 6). This finding corroborates the findings of Reddy et al. (2016), in which forest land was converted to agriculture, and increases in the plantations and submergence of forest due to the construction of reservoirs were identified as the major drivers of deforestation of the Western Ghats region.

#### Change in agricultural land

**Fig. 7:**
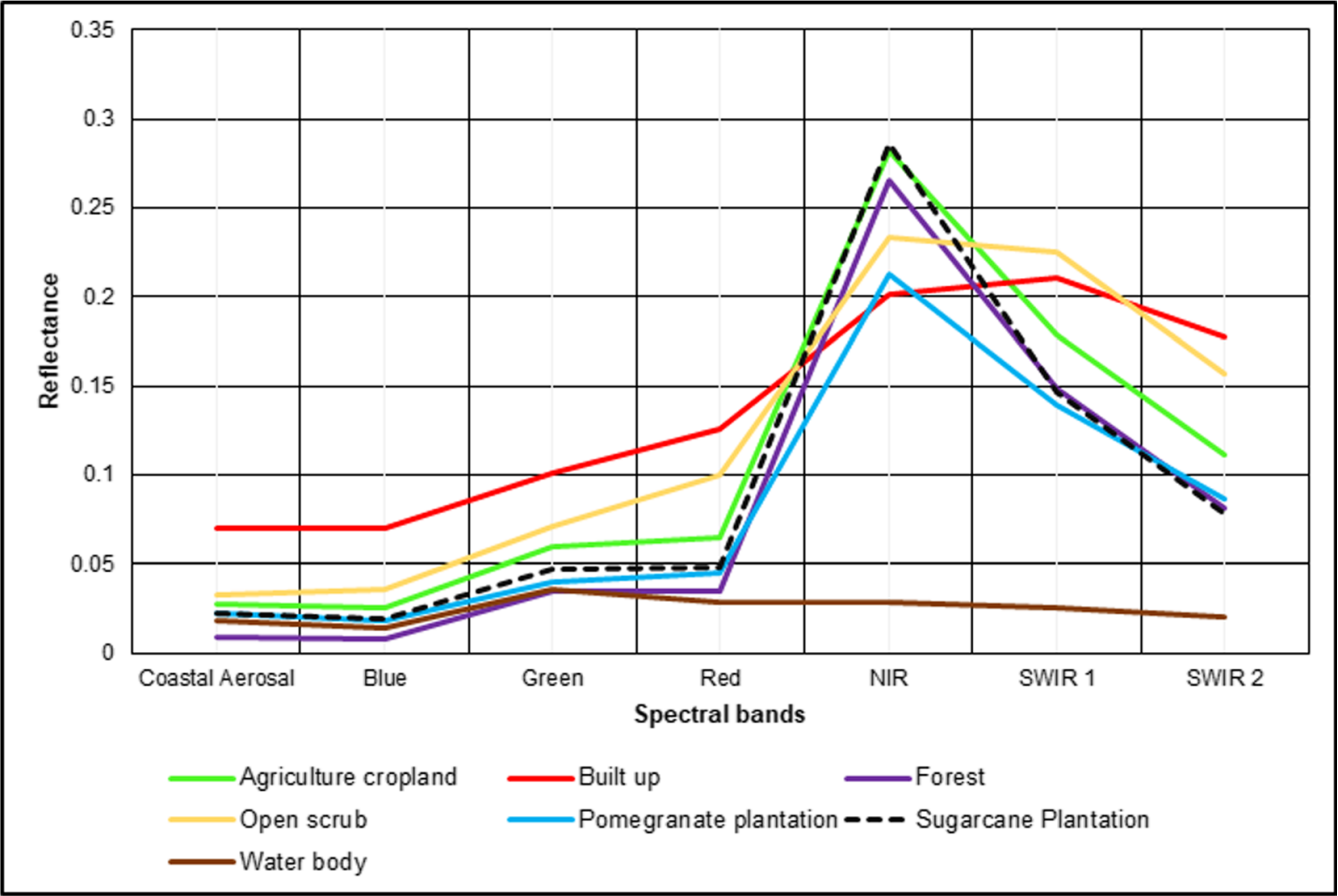
Landsat 8 imagery surface reflectance of various LULC classes

Reductions in the agricultural cropping intensity and vegetative cover resulted in a negative change in vegetation. A predominant shift was noticed from agriculture crops to plantations in the N3 and N4 locations. The increase in the area under plantations was primarily due to sugarcane and pomegranate cultivation. Pomegranate accounted for the major share of the increased area. Sugarcane cultivation was noted primarily in the command area of the Nilwande irrigation project. The negative change in vegetation can be attributed to pomegranate, which is a tropical shrub with less canopy cover. A reduction in the surface reflectance of pomegranate (Fig. 7) resulted in lower NDVI values and was attributed to a negative change in the vegetation canopy. In addition, the area under triple (multi-seasonal) cropping was converted into double-cropped agriculture.

The major drivers associated with pomegranate expansion could be government promotional schemes (namely, the NHM initiated in 2005), increased water access, and improved road networks. NHM is a Government of India-sponsored scheme that provides subsidies, technical support, and capacity-building activities for horticultural development (Kumar 2013). These subsidies had a positive influence on the pomegranate cultivation in this region (Pawar and Bhor 2012; Aher and Rahane 2016; Chavai and Kadam 2016). Access to groundwater is another important driver for the increased area under pomegranate plantations (e.g., all the pomegranate farmers at the sample GT points had access to groundwater through either dug wells or tube wells). In the Ahmednagar district, the percentage of groundwater irrigation relative to the net irrigated area increased from 68% in 1991 to 81% in 2012 (Mane 2012). The export of pomegranates increased rapidly over the last decade. Sangamner is well-connected by national and state highways that link to major urban centers such as Mumbai, which is a major business hub. Almost 53.7% of the pomegranate fruits were exported to the UAE in 2013, with total exports of 36,026 tons (Shinde et al. 2016). Higher profits from pomegranate cultivation prompted farmers to shift away from food crops in many of the villages in Mula-Pravara (Jadhav and Shinde 2015). In the Ahmednagar district, the area under pomegranates alone increased more than three times from 2010 to 2013 (Government of Maharashtra 2015). All these developments helped to increase the area under pomegranate and sugar cane plantations, from 121 km^2^ in 2011 to 325 km^2^ in 2016 (Table 4).

**Fig. 8:**
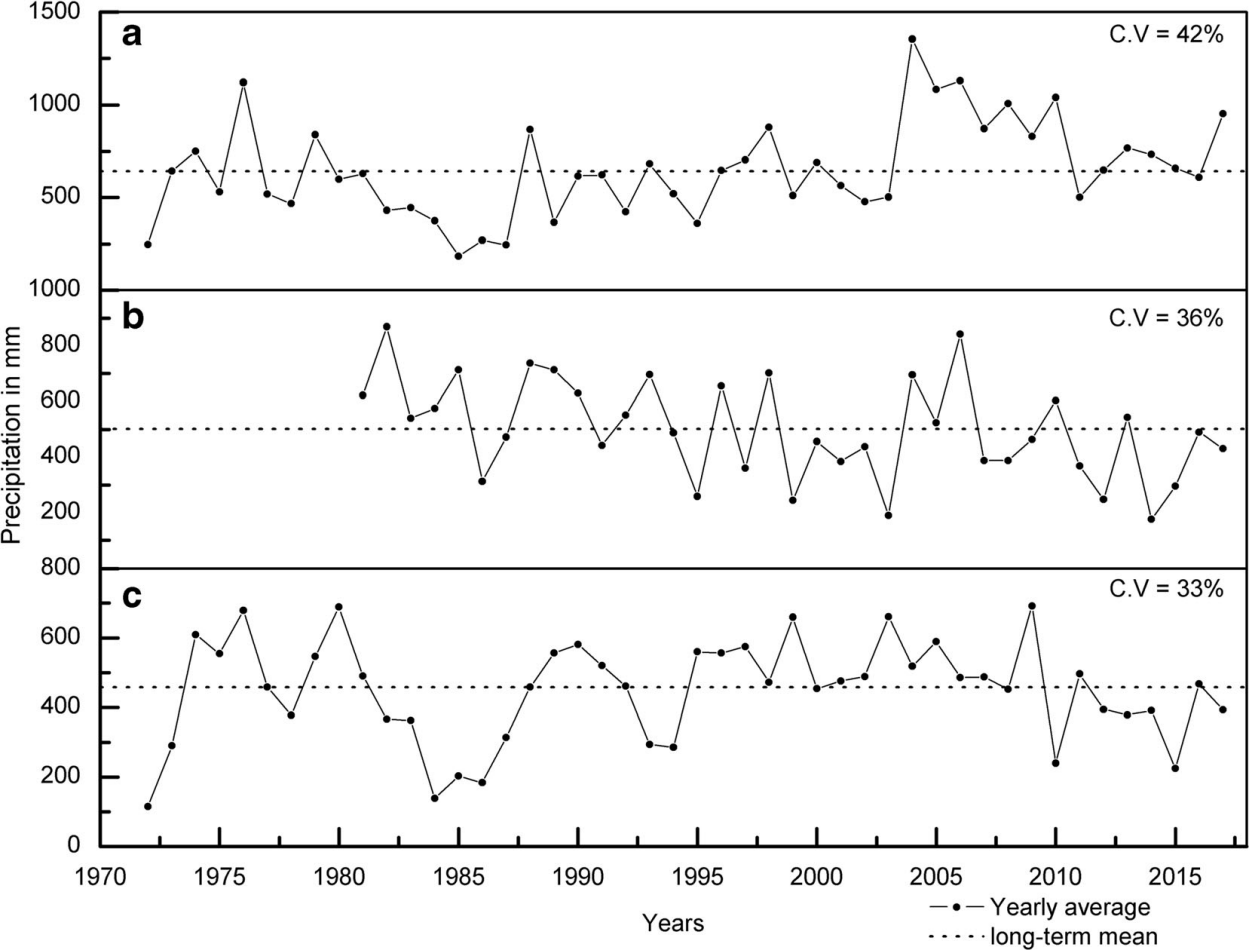
Annual mean rainfall of the study area (**a** Akole; **b** Parner; **c** Sangamner)

In both locations, there was a decline in the triple-cropped area (due to the reduced area under Zaid crops). This decline is primarily due to reduced annual rainfall from 2010 to 2015 in Sangamner (Fig. 8), where the N3 and N4 hotspots are located. In Sangamner, dug wells (that tapped into shallow aquifers) were a major source of irrigation. The decline in rainfall results in reduced surface flow and the recharge of shallow aquifers, which in turn leads to a water shortage for agriculture.

#### Change from agricultural to built-up land

The conversion of larger patches of agricultural land to the built-up category occurred in N4 (Fig. 6 N4), which is primarily due to the expansion of the Sangamner urban center.

**Fig. 9:**
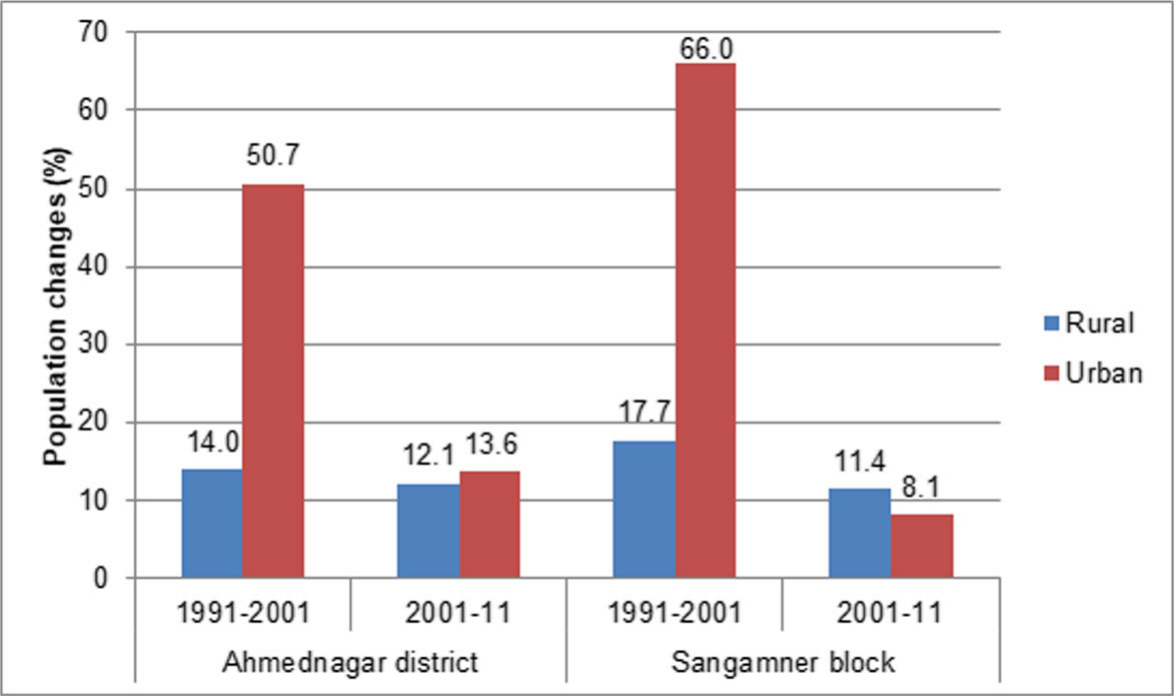
Percent change in the urban population from 1991 to 2011

Sangamner is the second-most populous city in the Ahmednagar district. The total population of Sangamner is 0.49 million (according to the 2011 census). The increase in the built-up area can be attributed to rapid growth in the urban population at both the district and block levels. From 1991 to 2001 (Fig. 9), the urban population growth in the Sangamner block (66%) was higher than that of the district (50.7%). This increase in population must have put pressure on the local land resources and could also be one reason for the conversion of agricultural land to settlements. The built-up class gained area mostly from wastelands from 1991 to 2001 and 2001–2011, whereas the conversion of agricultural to built-up land took place from 2011 to 2016 in the Sangamner city fringe areas of *Gunjalwadi*, *Dholewadi*, *Kasar Dumala*, and *Ghulewadi* villages (Fig. 6 N4).

### Positive change in vegetation

**Fig. 10:**
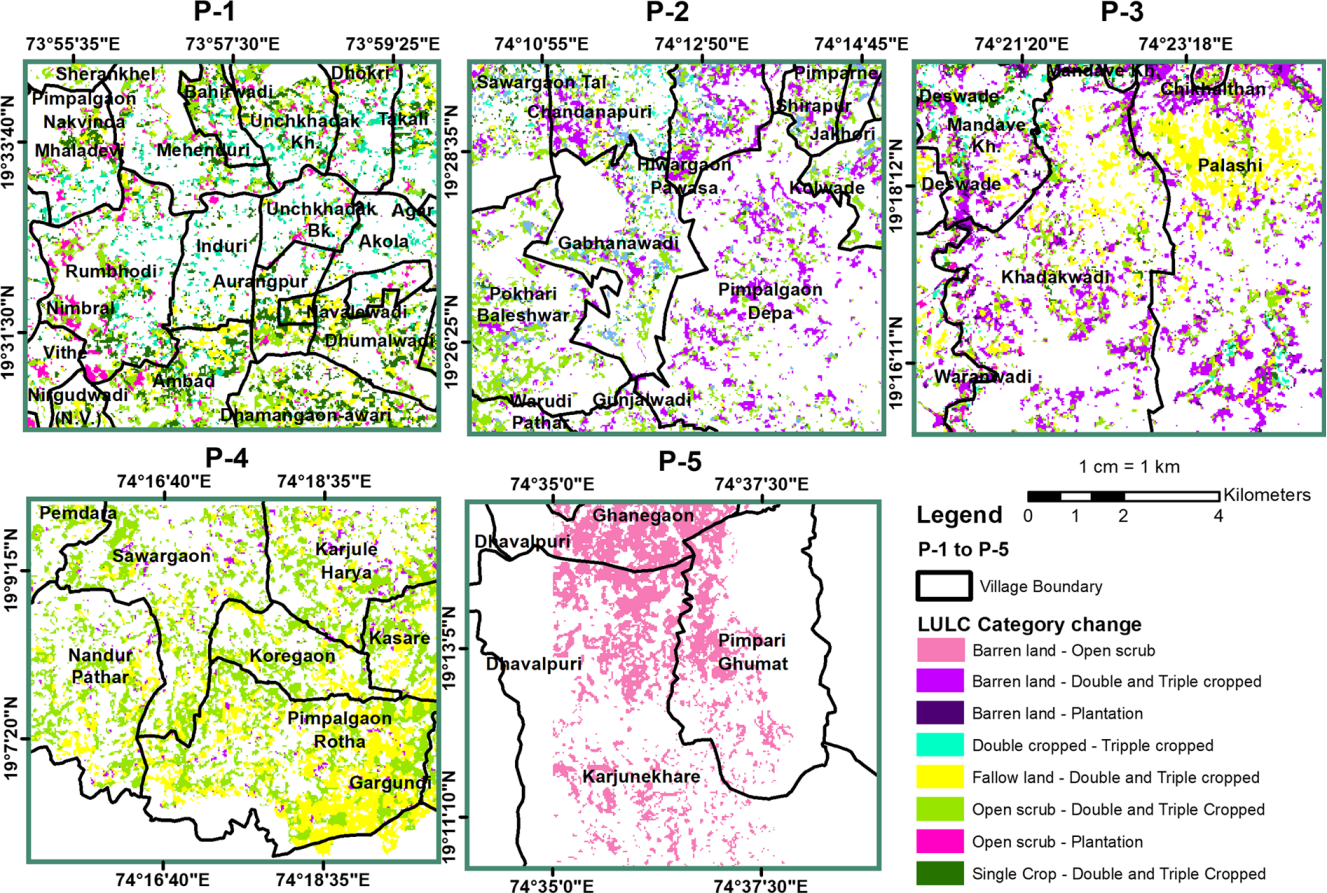
Hotspots of positive change, P1 to P4; increase in cropping intensity P5, increase in vegetative cover

The positive change in vegetation was found at five locations of P-1 (Akole block), P-2 (Sangamner block), P-3, P-4, and P-5 (in Parner block) (Fig. 10). Among the five positive hotspots, four (P1 to P4) showed increased cropping intensity (shifting from double to triple-cropping) and the conversion of UCW lands (open scrub, fallow lands, and barren lands) to agricultural land. In P5, the positive change is due to the increase in tree cover. In P-1, the positive change in vegetation was due to the conversion of croplands into sugarcane plantations, which resulted in increased reflectance in the spectral signature, and it showed a positive trend in NDVI (Fig. 7). In addition, there was also a change from open scrub to cultivated land. This change could be due to the Nilwande dam, which became operational in 2011, and it brought a total of 33,148 hectares (ha) of agricultural land under canal irrigation (Agriculture Census 2012). This development provided the opportunity to the farmers in this area to shift to water-intensive commercial crops such as sugarcane. In P2 (Fig. 10), the major increase in the cultivated area is due to access to groundwater, water-harvesting structures such as farm ponds and micro-irrigation schemes (drip irrigation).

In P-3, the watershed development programs (e.g., the integrated watershed development program) appeared to have helped increase the cultivated area and cropping intensity. Some parts of P-3 have also benefited from the “*Mandohol*” medium irrigation project. In P-4 (Parner), large areas of fallow land and open scrub were converted into agricultural land (Fig. 10). This conversion primarily occurred because of the increase in the irrigated area under wells/tube wells and tanks. For example, according to the census from 2011, in the village *Pimpalgaon Rotha* (in P-4), 53% of the total irrigated area was under wells/tube well irrigation and the remaining area was covered by tanks. P-5 showed increased vegetative cover in the upper catchment of the Mula River. The dense growth of natural vegetation was observed along the drainage lines, which could be due to the increased soil moisture because of the presence of water conservation structures.

### Drivers of land use and land cover change

The LULC changes were both positive and negative. In some hotspots, there was a shift from agricultural land to horticultural plantations (pomegranates or sugarcane), which are considered here as a negative change in agricultural land. There was also the conversion of agricultural land to the built-up area. The forest canopy area was found to decline. In other hotspots, there was positive change due to the conversion of fallow and open scrub into agricultural land. There has also been an increase in the cropping intensity. The major drivers of LUCC identified in the previous sections are summarized below. The drivers are grouped into institutional, economic, technological, and natural factors (Lambin et al. 2003).

#### Institutional factors

Institutional factors affected the LULC changes in this region, and they include government policies and programs, legal frameworks, and governance mechanisms (or lack thereof) and their interactions with individual decision makers. Investments in major and medium irrigation projects (for example, Pravara, Nilwande, and Mandohol), along with better access to groundwater in the region, increased the area under irrigation. In addition, the widespread promotion of watershed development programs by the government, external donor agencies, and NGOs in the early 90s (Farrington and Lobo 1997) resulted in extensive water and soil conservation measures, which increased the water-spread area in semi-arid regions (Shah 1998). This, in turn, secured water availability for the farmers in the area, enabling them to cultivate larger areas (Garg et al. 2012; Bhan 2013).

As of 2015, in Mula-Pravara basin, almost 38% (1615 km^2^) of the area has been covered by various watershed development programs (IWMP 2016). There are a number of examples from the region where watershed development programs increased the area under irrigation (Tilekar et al. 2009; Wani et al. 2011; Gray and Srinidhi 2013). Many government policies and schemes such as agricultural input subsidies (for seed, fertilizers, electricity, agricultural machinery, and micro-irrigation) also played an important role in agricultural development in the region (Dev and Mungekar 1996; Kerr 2002; Wani et al. 2011; Wani et al. 2016). For example, the increased area under pomegranates in this region is an outcome of this support through various horticulture schemes (Chavai and Kadam 2016).

Groundwater, which was one of the key drivers of change in agriculture, became an overexploited resource in this region (Central Groundwater Board 2017) due to limited groundwater governance mechanisms (Kulkarni et al. 2015). In addition, the recently enacted Maharashtra groundwater (development and management) act from 2009 is facing implementation issues due to the poor institutional framework (Joshi and Aslekar 2018).

#### Economic factors

Improved access to markets influenced the cropping pattern in the region. The share of commercial crops in the total cultivated area has increased in the Ahmednagar district (Rede et al. 2013). The shift has moved towards crops such as pulses, cotton, sugarcane, and soybeans. Market opportunities due to the export potential interacted with institutional factors (government subsidies, NHM) to establish pomegranates as a profitable plantation crop in recent times. Farmer income almost doubled from pomegranate cultivation, and the reported annual income was between INR 400,000 to 700,000 (Jadhav and Shinde 2015). In recent years, pomegranate exports to foreign countries have increased five-fold, and in 2013, the total export was 36,026 tons (Shinde et al. 2016). In the case of sugarcane, the presence of sugar factories in the local area provided immediate market opportunities for the farmers in nearby villages. The Ahmednagar district has the highest number of sugar factories in the state of Maharashtra (with 18 sugar factories). The area under sugarcane cultivation has almost doubled in the district, from 77,787 ha in 1990–1991 to 147,215 ha in 2010–2011 (Agriculture census 2012). The growth in many small and large-scale industries increased the employment opportunities in Sangamner city (Shantaram 2009). This activity could have triggered the growth of the urban population witnessed in this region, which in turn could have led to the conversion of agricultural land into built-up areas (Fig. 6).

#### Technological factors

The advent of tube well technology in the mid-60s made groundwater the major source of irrigation in the area, giving farmers an opportunity to bring fallow and scrublands under cultivation (Dhawan 1979). However, this advance also resulted in an overexploitation problem due to the existing groundwater usage pattern (the conversion of traditional water-harvesting structures such as farm ponds to groundwater storage structures) (Kale 2017). Agricultural mechanization, high-yielding crop varieties and fertilizer use have enhanced the Net Primary Productivity (NPP) in agriculture in this region (Gholkar et al. 2014). Similar observations were made regarding the role of agricultural technologies that resulted in an increased area under cultivation in the country (Tian et al. 2014; Roy et al. 2015; Meiyappan et al. 2016). In the case of pomegranate cultivation, the development of new varieties that are high-yielding and tolerant to stressors (climatic and pests) complemented the growth (Chandra and Tarachand 2010). Micro-irrigation technology was encouraged through government schemes (under the National Mission on micro-irrigation and the National Mission on Sustainable Agriculture, “Pradhan Mantri Krishi Sinchai Yojana” scheme), with a total outlay of INR 57.89 billion between 2009 and 2015 (Kapur et al. 2016). All these approaches resulted in a larger number of farmers in the area adopting these technologies, thereby improving the water use efficiency (Narayanamoorthy 2004).

#### Natural factors

This region experiences high rainfall variability (Fig. 8), prolonged dry spells (TERI 2014), and frequent droughts (Gupta et al. 2011). In addition, the geology of Mula-Pravara consists of basaltic hard rocks, which are part of the Deccan traps. Groundwater is the major irrigation source, and only approximately 10% of the agriculture area is covered under surface irrigation schemes (Mane 2012). The availability and occurrence of groundwater is not infinite in the area, and it is restricted only along weathered zones, fractured zones, and joining patterns of trap rocks (Limaye 2010; Thomas and Duraisamy 2016). Due to the higher groundwater dependence, almost 34% of the basin area has already been classified as an “overexploited” groundwater zone (MWRRA 2015). Therefore, a rapid decline in groundwater resources makes this region vulnerable to future risks.

## Conclusions

This study provides a description of the finer scale LUCC pattern and locations of significant change (hotspot), which is a valuable source of information for explaining regional-level changes in the semi-arid area, and it is useful for predicting future land use development in the Mula-Pravara region. It was observed that there was a significant positive change in the area under agriculture from 1991 to 2016. There has been an increase in the agricultural land area by approximately 98%. The cultivated area under the Rabi season increased by approximately 60% from 2011 to 2016. The area under plantations also increased dramatically, by 1601%. Simultaneously, there was a decline in the UCW category by 34.6% and in fallow lands by 60.5%. All these changes could be largely attributed to farmer access to groundwater, irrigation projects, and watershed development programs, which resulted in the conversion of hitherto fallow lands and wastelands into cultivated area.

Groundwater is considered as an open access/common pool resource, and it played a major role in the agricultural growth in the region. However, groundwater is not an infinite resource, and it has its own limitations in terms of recharge potential. For example, in the present study region, the local geology consisting of hard rock basalt severely limits groundwater availability and recharge potential. Groundwater over-exploitation and mismanagement is a common characteristic across all the semi-arid regions in India (Kumar et al. 2012; Kulkarni et al. 2015). Therefore, overdependence on groundwater could have serious implications for sustaining the agriculture in the region. It is important to prioritize groundwater management by implementing the recently enacted Maharashtra Groundwater (Development and Management) Act of 2009 to regulate groundwater (MWRRA 2013), which is a first step towards addressing the governance issues surrounding groundwater use.

Access to markets and government incentives have provided opportunities for farmers to cultivate high input and high-profit commercial crops. Farmers are exposed to both market and climate risks. Therefore, it is important to enhance agro-advisory systems and the information technology infrastructure to make market, climate, and crop management information accessible to farmers and to develop appropriate risk mitigation strategies. The degradation of dense forest canopies to open forest and scrub forest has been observed in the protected areas of the WG region. To strengthen the governing mechanisms to manage forests, it is imperative to engage the local communities actively in planning and monitoring activities to optimize the use of ecosystem services (fodder, non-timber forest products, and firewood) and prevent illicit tree cutting. Managing forests well would assist in maintaining the surface and sub-surface water flows, reduce soil erosion, and enhance carbon sequestration.

The built-up area increased by 195% from 1991 to 2016. In recent times, more of the agricultural lands in the fringe areas of urban centers have been converted into built-up areas. This unplanned growth and the expansion of urban centers are causes for concern, which puts pressure on the land and water resources in the peri-urban regions. Future climate risks in terms of dry spells, rainfall variability, and increases in the frequency of drought incidences further aggravate the vulnerability of the communities in the region.

Further research using contemporary optical and microwave Sentinel series satellite data would help to generate information on the spatial distribution and acreage of cultivars. These data could be of potential use in developing location and crop-specific advisories as well as enhancing the water resource management in this region.
